# Comparative Effectiveness of Remestemcel-L-rknd versus Ruxolitinib in Pediatric Patients with Steroid-Refractory Acute Graft-Versus-Host Disease using Simulated Treatment Comparisons

**DOI:** 10.36469/jheor.2021.19008

**Published:** 2021-02-23

**Authors:** Gabriel Tremblay, Dimitrios Tomaras, Eric Strati, Anna Forsythe

**Affiliations:** 1 Purple Squirrel Economics, New York, NY; 2 Mesoblast Inc., New York, NY

**Keywords:** graft-versus-host-disease, pediatric, comparative effectiveness, simulated treatment comparison

## Abstract

**Background:** Allogeneic hematopoietic stem cell transplantation (allo-HSCT) can be a lifesaving treatment for hematologic malignancies, but acute graft-versus-host-disease (aGVHD) is a potentially deadly adverse effect experienced by up to half of allo-HSCT recipients. Inadequate response to steroid therapy for aGVHD is associated with poor prognosis and high mortality, including among pediatric patients, who are the focus of this study. Ruxolitinib and remestemcel-L-rknd were evaluated for the treatment of steroid-refractory (SR) aGVHD in two separate single-arm trials. To effectively compare the safety and efficacy of these treatments without a head-to-head trial, a simulated treatment comparison (STC) was conducted.

**Methods:** Regression techniques were used to adjust individual patient-level data from the remestemcel-L-rknd trial to mutually reported baseline characteristics from the ruxolitinib trial. Outcomes of interest included a 28-day overall response rate (ORR), a 28-day ORR in the grade III-IV aGVHD population, and adverse events (AEs).

**Results:** In the full populations, the STC of risk ratios (RRs) found treatment with remestemcel-L-rknd to be associated with a numerical but not statistically significant improvement in the 28-day ORR versus ruxolitinib. In the grade III-IV aGVHD sub-group, the STC showed significantly improved 28-day ORR for remestemcel-L-rknd versus ruxolitinib (*P*=0.04). Remestemcel-L-rknd was also associated with improved safety outcomes (*P*<0.05) in 17 out of 30 AEs, including hematologic events, peripheral edema, muscular weakness, nausea, back pain, and fatigue.

**Conclusion:** Remestemcel-L-rknd was associated with significant improvements in day 28 ORR compared with ruxolitinib in patients with severe (grade III-IV) SR aGVHD. Across all grades of SR aGVHD, remestemcel-L-rknd was associated with fewer all-grade treatment-emergent adverse events (TEAEs) (27/30) available for comparison, including the majority reaching statistical significance.

## BACKGROUND

Allogeneic hematopoietic stem cell transplantation (allo-HSCT) is a potentially curative treatment for children with hematological malignancies and other indications such as sickle cell disease or aplastic anemia.^1–3^ In 2016, 1771 allo-HSCT procedures were performed in pediatric patients in the United States (US).^4^ Graft-versus-host-disease (GVHD), which can manifest as acute or chronic disease, is a serious and life-threatening complication of allo-HSCT. Acute GVHD (aGVHD) generally occurs within 100 days of treatment, whereas chronic GVHD, which can follow aGVHD, typically has a later onset and may persist over the long term.^5^

GVHD is a rare orphan disease with a prevalence of one to nine per 100 000 persons.^6,7^ Acute GVHD occurs in 35% to 50% of all allo-HSCT recipients; in 2016, there were about 38 000 allo-HSCTs performed globally, i.e., an estimated 13 300 to 19 000 patients worldwide may have developed aGVHD.^7,8^ Severe grade III to grade IV GVHD is associated with poorer outcomes and occurs within 100 days of treatment in 16% of allo-HSCT recipients with sibling donors and 32% of recipients with unrelated donors.^7,9^ Patients have a higher risk of aGVHD when they have a greater degree of human leukocyte antigen (HLA)-mismatch between the donor and recipient.^1^

Patients who develop aGVHD following allo-HSCT are at risk of post-transplant non-relapse-related mortality.^10^ Mortality associated with aGVHD has historically been reported to range from 70% to 90% in adults and children who fail to respond to initial steroid therapy, although survival has improved with advances in supportive care.^11–13^ In a single-center study among children with steroid-refractory aGVHD (N=240), the one-year mortality rates were 50%.^14^ In addition to increased mortality risk, aGVHD in pediatric patients is associated with extensive health-care costs.^15^ A US study of pediatric patients with GVHD reported an average increase in health-care costs of US$173 836 and an average increase of 45.4 days in inpatient length of stay in pediatric allo-HSCT patients who developed aGVHD compared with those who did not (*P*<0.001 for both comparisons).^14^ In a retrospective cohort study (N=240), the median one-year inpatient and outpatient costs in pediatric patients with grade >1 aGVHD have been estimated at US$224 000.^14^ Development of aGVHD in pediatric patients (n=26) is associated with a seven-fold greater risk of increased inpatient costs (odds ratio [OR] 7.11, 95% confidence interval [CI] 1.02-49.46; *P*=0.047).^16^ Furthermore, a recent claims analysis showed that pediatric patients with steroid-refractory aGVHD (n=38) are estimated to incur average additional health-care costs of US$500 000 over 12 months compared to controls without GVHD (n=184).^17^ In addition to the clinical and economic burden, acute and chronic GVHD in pediatric patients is associated with a considerable impact on the quality of life of patients and their caregivers.^18–20^

Corticosteroids are the first-line treatment for aGVHD, according to the American Society of Blood and Marrow Transplantation;^21^ however, less than 50% of patients achieve a sustained response after one week.^22^ Immunosuppressive therapies are used in the second line, but there is limited evidence on their efficacy in children or adults with aGVHD.^2^

Ruxolitinib is a Janus kinase (JAK) inhibitor that was approved by the US Food and Drug Administration (FDA) in May 2019 for the treatment of steroid-refractory aGVHD in patients aged ≥12 years.^23,24^ FDA approval was based on a phase 2 single-arm trial (REACH-1) in 49 adults aged ≥18 years with aGVHD refractory to steroids alone, with additional bioequivalence, pharmacokinetic and safety data in children to support its use in the pediatric patient population ≥12 years old.^23^ Prior to ruxolitinib, there were no FDA-approved therapies specifically for the treatment of aGVHD. Remestemcel-L-rknd (Mesoblast, Australia) is a human mesenchymal stromal cell therapy that has been studied specifically in pediatric patients (N=55) with steroid-refractory aGHVD in a single-arm phase 3 clinical trial (NCT02336230).^25,26^ Among the 54 children who received treatment with remestemcel-L-rknd, the 28-day overall response rate was 70.4%, with 78.9% survival at day 180 for responders, versus 43.8% survival for nonresponders (*P*=0.003).^26^

In an evolving treatment landscape, it is possible that clinical and reimbursement stakeholders will make treatment decisions across various therapies based on limited comparative evidence. There are no head-to-head studies comparing ruxolitinib with remestemcel-L-rknd. Owing to the severity and rarity of pediatric aGVHD and the ethical issues associated with placebo-controlled trials in this indication, most clinical trials typically have been small and uncontrolled, and single-arm in design. For clinical and reimbursement decision-makers to conduct robust comparisons, population differences (e.g., patient demographic and disease characteristics) as well as clinical safety and efficacy outcomes between the pivotal trials would have to be considered.

We contrasted the efficacy and safety profiles between the two pivotal single-arm phase 2 (ruxolitinib) and phase 3 (remestemcel-L-rknd) studies using a statistical-adjustment method to adjust for imbalances in patient characteristics.

## METHODS

**Figure 1.**
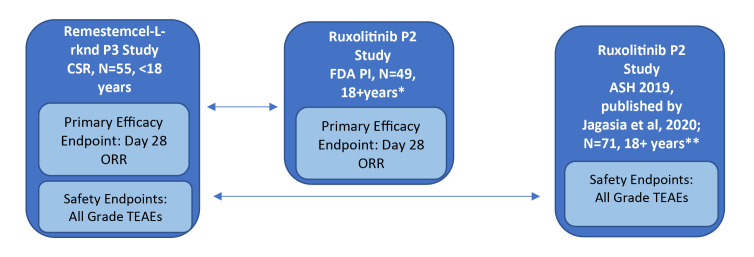
Clinical Studies and Data Sources for Comparative Effectiveness Analysis Indirect and Simulated Treatment Comparison Approaches Abbreviations: ASH, American Society of Hematology; CSR, clinical study report; FDA, Food and Drug Administration; ORR, overall response rate; PI, prescribing information; P3, phase 3; TEAEs, treatment emergent adverse events. *patients refractory to steroids alone. **patients refractory to steroids with or without other immunosuppressive drugs. Adapted from: Mesoblast, data on file, 2019a and 2019b^27,28^; Jagasia et al., 2018^24^; Incyte, 2019^23^; Jagasia et al, 2020^29^

The study designs of the phase 2 single-arm ruxolitinib trial (REACH-1) and the phase 3 single-arm remestemcel-L-rknd trial (NCT02336230) were evaluated by two independent researchers and found to have only minor differences. As both trials were single arm in design, there was no common comparator arm to serve as an anchor; only unanchored comparisons in the two active treatment groups were possible. Individual patient-level data for the phase 3 remestemcel-L-rknd trial were obtained from the clinical study report (data on file).^27,28^ Safety and efficacy data for remestemcel-L-rknd were reported as treatment-emergent adverse events (TEAEs) and overall response rate (ORR) at day 28, respectively. However, the adverse event data presented in the ruxolitinib FDA prescribing information were not specifically defined as TEAEs, necessitating the use of two separate data sources for the same REACH-1 trial to obtain equivalent safety and efficacy data to enable comparisons with remestemcel-L-rknd. Compatible efficacy data for ruxolitinib in patients refractory to steroids alone were reported in the FDA package insert (N=49), whereas compatible TEAEs safety data were reported in the oral presentation at the American Society of Hematology (ASH) conference and later published for patients steroid refractory alone (n=49) and patients refractory to steroids plus other immunosuppressive drugs (n=22), (N=71, full REACH-1 population).^24,29^ The efficacy and safety data sources for the ruxolitinib and remestemcel-L-rknd clinical studies are presented in Figure 1.

### Indirect and Simulated Treatment Comparison Approaches

As both data sources were from single-arm clinical trials, anchored comparison was unfeasible; unanchored techniques were necessary to compare outcomes (indirect treatment comparison [ITC], matching adjusted indirect comparison [MAIC], and simulated treatment comparison [STC]). As MAIC uses propensity weighting scores, sample sizes are reduced significantly; the National Institute for Health and Care Excellence (NICE) Decision Support Unit report on population-adjusted indirect comparisons provided an example in which, after evaluating several published MAIC analyses, concluded that weighting reduced effective sample size by an average of 80% (range 57% to 98%); this can lead to inferences dependent on very low numbers of patients when starting from relatively small studies.^30^ Given that the ruxolitinib and remestemcel-L-rknd trials selected for analysis included small patient populations (49 and 55 patients, respectively), MAIC techniques substantially reduced the sample size of an already small population and thus were deemed inappropriate for analysis. STC analysis uses regression-based techniques, incorporating the entire patient data set, without any patient loss. As the size of the sample populations was already limited, STC was considered the most appropriate technique for an unanchored comparison. Limitations pertaining to this population adjustment methodology are noted in the Discussion section of this manuscript.

Methodology was based on the NICE Decision Support Unit’s Technical Support Documents for population-adjusted indirect comparisons (MAIC and STC) to ensure robustness and transparency.^30^ A full description of STC and standard unadjusted indirect treatment comparison (ITC) is available in the literature and in the NICE Technical Support Documents.^30–32^ In brief, logistic regression outcome models with a log link were constructed for ORR at day 28 using the individual patient-level data of the remestemcel-L-rknd phase 3 study. Mutually available patient characteristics that demonstrated either prognostic association with outcomes or effect modification with treatment were considered for inclusion. Prognostic variables were identified based on statistical testing using univariate logistic regression models (results shown in Table 1). Effect modifier status of covariates was validated through literature, reviewed by key aGVHD clinical experts, and carefully monitored to ensure that the elimination process did not remove any significant effect modifiers. Following clinically guided backwards stepwise elimination, Chi-square, log likelihood and Akaike information criterion/Bayesian information criterion statistics were used to determine the optimal model.

**Table 1.** Mutually Reported Baseline Characteristics

**Variable**	**Remestemcel-L-rknd** **N=55**	**Ruxolitinib** **N=49**	ORR *P*-Value**	**ORR Final Model Rationale**
**Age (years)**			0.98	Omitted due to difference in age inclusion
Median	7	57	criteria	
Range	<1-17	18-72		
**Sex**			0.39	Included-strongly imbalanced and potential effect modifier by key aGVHD clinical experts
Male (%)	64%	47%		
**Ethnicity**		0.81		Included-strongly imbalanced and potential effect modifier by key aGVHD clinical experts
White (%)	56%	92%		
Other (%)	44%	8%		
**Baseline aGVHD grade** (%)*		0.40		Included-strongly imbalanced and potential effect modifier by key aGVHD clinical experts
II	11%	27%		
III	42%	55%		
IV	47%	18%		

Abbreviations: aGVHD, acute graft versus host disease; ORR, overall response rate.

*For remestemcel-L-rknd, aGVHD grading is presented as per International Bone Marrow Transplant Registry (IBMTR) classification, whereas ruxolitinib grading is presented using the Mount Sinai Acute GVHD International Consortium (MAGIC) classification.

**Based on univariate logistic regression for day 28 ORR

Source: Adapted from Mesoblast, data on file, 2019^27^; Jagasia et al., 2018.

In addition to STC, outcomes were also compared using ITC techniques unadjusted for baseline characteristics. ITC was used in this case for reference to quantify the magnitude of the effect of STC covariate adjustment. To do this, unadjusted ITC techniques were used to produce RRs with 95% CIs and *P*-values for each outcome, and adjusted STC was conducted similarly for ORR at day 28. Equation 1 was used to estimate the indirect risk ratio (RR) based on unadjusted and adjusted outcome proportions from the index and comparator trials. Differences were deemed statistically significant at *P*-value ≤0.05.

**Equation 1.** RR = (ni/Ni)/ (nk/Nk), where n is the outcome counts, and N is the total sample sizes of the index trial (i) and comparator trial (j).

### Statistical Adjustments

All analysis was performed using Stata^®^ software (StataCorp. 2019. Stata Statistical Software: Release 16. College Station, Texas: StataCorp LLC) and Microsoft^®^ Excel^®^ (v16.0, 32-bit). For STC analyses, stepwise logistic regression was used to adjust for mutually reported patient covariates, adjusting patient-level data to available ruxolitinib data.

Key assumptions included the independence of baseline characteristics, thus minimizing the effect of collinearity among covariates. The stepwise model for ORR at day 28 included covariate adjustment for the following mutually reported baseline characteristics: ethnicity (Caucasian), aGVHD grade, and sex. Table 1 summarizes mutually available baseline characteristics, *P*-values for ORR, and stepwise model inclusion justification.

Prior to adjustment, using patient-level data from the remestemcel-L-rknd trial, aGVHD grade was reclassified to Mount Sinai Acute GVHD International Consortium (MAGIC) Criteria to match the grades reported in the REACH-1 ruxolitinib trial.^24^ Because the youngest patient in the ruxolitinib trial was 18 years old and the oldest patient in the remestemcel-L-rknd trial was <18 years old, statistical adjustment for age was not feasible. Despite the difference in age range, it was therefore assumed that age was not associated with outcomes. We acknowledge this as an essential assumption that may represent a limitation of the analysis; however, comparable outcomes across age groups in steroid-refractory aGVHD have been noted in the literature [Rashidi 2019; MacMillan 2019]. Furthermore, the idea that aGVHD outcomes are not strongly associated with age is suggested by the recent FDA approval of ruxolitinib in steroid-refractory aGVHD in patients ≥12 years and <18 years old on bioequivalence data only. Given the modest number of relatively small studies in SR aGVHD that were not powered to assess differences among age groups, the impact of age on outcomes in SR aGVHD is not fully understood. Lastly, there is a practical need for reimbursement decision-makers to compare treatments using the most robust methods available.

### Outcomes Assessed

The primary efficacy outcome for comparison in this study was day 28 ORR. Additionally, day 28 ORRs were calculated and compared for the subpopulation of patients with more severe disease (grade III-IV aGVHD). Safety outcomes were also calculated for individual TEAEs reported in both studies. Owing to the limited number of patients in both trials with patient-level TEAE data, adjustment for baseline characteristics was not feasible for TEAEs.

## RESULTS

### Patient Characteristics

There were key differences in the patient baseline characteristics between the two trial populations in age, sex, ethnicity, and disease severity.

Patients in the remestemcel-L-rknd study were younger, aged between <1 to 17 years (median of 7 years), whereas the ruxolitinib study included older patients aged 18 to 72 years (median of 57 years). At baseline, the proportion of males in the remestemcel-L-rknd study was higher than the ruxolitinib study (64% versus 47%). Approximately half of the patients in the remestemcel-L-rknd study were white, compared with nearly all patients in the ruxolitinib study (56% versus 92%). In addition, a greater proportion of the remestemcel-L-rknd patient population had more severe disease (grade III-IV) at baseline compared with the ruxolitinib patient population (89% versus 73%). Patient baseline characteristics from the two ruxolitinib and remestemcel-L-rknd studies are presented in Table 1.

### Efficacy – Day 28 ORR

While there are no head-to-head data, by naïve comparison, the day 28 ORR was higher with remestemcel-L-rknd at 70.4%, compared with an ORR of 57.1% for ruxolitinib in the REACH-1 study, as reported in the FDA package insert.^23,27^

**Figure 2.**
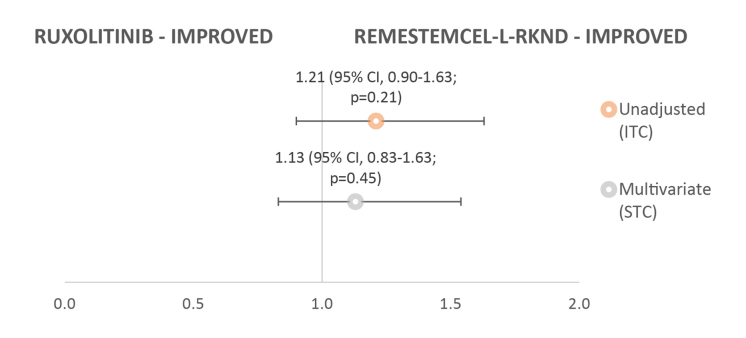
Risk Ratio for Day 28 ORR of Remestemcel-L-rknd (N=55) Versus Ruxolitinib (N=49) Abbreviations: ITC, indirect treatment comparison; ORR, overall response rate; STC, simulated treatment comparison.

Using the full population set, a trend toward day 28 ORR improvements with remestemcel-L-rknd was observed; however, the difference was not statistically significant in both adjusted (STC) and unadjusted (ITC) analyses (Figure 2). The RR of remestemcel-L-rknd versus ruxolitinib for day 28 ORR was 1.21 (95% CI, 0.90-1.63; *P*=0.21) without any adjustment for covariates, and 1.13 (95% CI, 0.83-1.63; *P*=0.45) following multivariate adjustment for baseline covariates.

**Figure 3.**
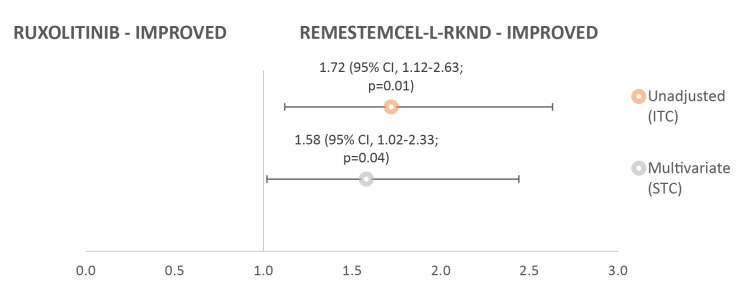
Risk Ratio for 28-day ORR of Remestemcel-L-rknd Versus Ruxolitinib in the Grade III-IV aGVHD Subgroup (N=49 for remestemcel-L-rknd and N=36 for ruxolitinib) Abbreviations: aGVHD, acute graft versus host disease; ITC, indirect treatment comparison; ORR, overall response rate.

In the subgroup of patients with severe aGVHD (grade III-IV) at baseline, day 28 ORR outcomes were statistically significantly in favor of remestemcel-L-rknd (Figure 3). The RR for remestemcel-L-rknd versus ruxolitinib was 1.72 (95% CI, 1.12-2.63; *P*=0.01) in unadjusted analyses, and 1.58 (95% CI, 1.02-2.33; *P*=0.04) in multivariate-adjusted analyses.

The day 28 ORR efficacy outcomes for remestemcel-L-rknd versus ruxolitinib for the full patient population and the severe aGVHD subgroup are presented in Table 2. Among the grade III-IV patients, ORR was significantly greater in remestemcel-L-rknd versus ruxolitinib.

**Table 2.** Efficacy: 28-day ORR for Remestemcel-L-rknd Versus Ruxolitinib

**Treatment comparison**	**RR**	**95% CI**	***P*-value**
Naïve comparison: ORR of 70.4% for remestemcel-L-rknd versus 57.1% for ruxolitinib			
ITC (unadjusted)	1.21	0.90-1.63	0.21
STC (adjusted)	1.13	0.83-1.63	0.45
ITC (unadjusted, grade III-IV aGVHD)	1.72	1.12-2.63	0.01
STC (adjusted, grade III-IV aGVHD)	1.58	1.02-2.33	0.04

Abbreviations: aGVHD, acute graft versus host disease; CI, confidence interval; ITC, indirect treatment comparison; ORR, overall response rate; RR, risk ratio; STC, simulated treatment comparison.

### Safety

In their respective trials, adverse events (AEs) leading to discontinuation were reported in 15% of patients treated with remestemcel-L-rknd and 31% of patients treated with ruxolitinib.^26,33^ The three most commonly reported TEAEs (all grades) with remestemcel-L-rknd were pyrexia, abdominal pain and adenovirus infection, reported in 33.3%, 20.4% and 20.4% of patients, respectively. The three most common TEAEs with ruxolitinib were anemia, hypokalemia and decreased platelet count, reported in 64.8%, 49.3%, and 45.1%, respectively (Table 3). Comparisons were conducted for individual TEAEs for which outcomes were reported in both studies. Overall, fewer TEAEs were observed with remestemcel-L-rknd than with ruxolitinib for most TEAEs (27 out of 30). Within the simulated trial comparison model, using unadjusted ITC techniques, the risk of a TEAE occurring (RR) was significantly lower with remestemcel-L-rknd compared with ruxolitinib for 17 out of 30 TEAEs (RRs with *P*≤0.05), as shown in Table 3.

**Table 3.** All-grade TEAEs of Remestemcel-L-rknd Versus Ruxolitinib

**TEAE**	Remestemcel-L-rknd* N=55	Ruxolitinib** **N=71**	**Unadjusted RR**	**95% CI**	***P*-value**
Anemia	7.4%	64.8%	0.11	0.04-0.30	<0.01
Hypokalemia	13.0%	49.3%	0.26	0.13-0.55	<0.01
Edema Peripheral	13.0%	45.1%	0.29	0.14-0.60	<0.01
Platelet Count Decreased	3.7%	45.1%	0.08	0.02-0.33	<0.01
Neutrophil Count Decreased	3.7%	39.4%	0.09	0.02-0.38	<0.01
WBC Decreased	3.7%	29.6%	0.13	0.03-0.51	<0.01
Muscular Weakness	1.9%	33.8%	0.05	0.01-0.39	<0.01
Fatigue	1.9%	29.6%	0.06	0.01-0.45	0.01
Nausea	5.6%	31.0%	0.18	0.06-0.57	<0.01
Vomiting	18.5%	25.4%	0.73	0.37-1.45	0.37
Diarrhea	13.0%	28.2%	0.46	0.21-1.01	0.05
Abdominal Pain	20.4%	21.1%	0.97	0.48-1.93	0.93
ALT Increased	7.4%	25.4%	0.29	0.10-0.81	0.02
AST Increased	1.9%	23.9%	0.08	0.01-0.56	0.01
Hyperglycemia	14.8%	25.4%	0.58	0.27-1.24	0.16
Hypomagnesemia	11.1%	32.4%	0.34	0.15-0.78	0.01
Hypophosphatemia	7.4%	25.4%	0.29	0.10-0.81	0.02
Back Pain	3.7%	23.9%	0.15	0.04-0.64	0.01
Acute Kidney Injury	7.4%	22.5%	0.33	0.12-0.93	0.04
Hypertension	18.5%	22.%	0.82	0.41-1.67	0.60
Hypotension	14.8%	21.1%	0.70	0.23-1.53	0.38
Headache	7.4%	21.1%	0.35	0.12-1.00	0.05
Pyrexia	33.3%	21.1%	1.58	0.88-2.84	0.13
Sepsis	5.6%	12.7%	0.44	0.13-1.55	0.20
Enterococcal Infection	3.7%	8.5%	0.44	0.09-2.08	0.30
Urinary Tract Infection	7.4%	8.5%	0.87	0.26-2.94	0.84
BK Virus Infection	13.0%	7.0%	1.86	0.62-5.53	0.27
Pneumonia	5.6%	7.0%	0.80	0.20-3.20	0.77
Staphylococcal Infection	9.3%	7.0%	1.33	0.40-4.36	0.65
CMV Infection	5.6%	14.1%	0.40	0.11-1.37	0.15

Abbreviations: ALT, alanine aminotransferase; AST, aspartate aminotransferase; CI, confidence interval; CMV, cytomegalovirus; TEAE, treatment emergent adverse event; RR, risk ratio; WBC, white blood cell.

Source: *Mesoblast, data on file, 2019; **Jagasia et al, 2018

Using the unadjusted ITC approach, lower risk was observed for ruxolitinib for pyrexia (RR 1.58; 95% CI, 0.88-2.84), BK virus infection (RR 1.86; 95% CI, 0.62-5.53), and staphylococcal infection (RR 1.33; 95% CI, 0.40-4.36) compared with remestemcel-L-rknd, but results were not statistically significant (*P*>0.05 for all comparisons).

## DISCUSSION

Steroid-refractory aGVHD in children is clinically challenging and costly, and it impacts patients, caregivers, and their families’ quality of life.^15^ Ruxolitinib is a JAK inhibitor that received FDA approval in May 2019 for the treatment of steroid-refractory aGVHD in patients 12 years old and older.^23^ In January 2020, the sponsor of remestemcel-L-rknd submitted the Biologic License Application for steroid-refractory aGVHD in the pediatric population to the FDA, with the FDA decision pending as of January 2021.^25^

There is no direct comparative data on safety and efficacy in this disease area. Owing to the severity and rarity of the disease, as well as the ethical and operational challenges enrolling these very sick patients, clinical trials in aGVHD are generally uncontrolled and include a small number of subjects rather than having a randomized, placebo-controlled trial design. Given the lack of direct comparative trials, robust statistical methods are required to enable reimbursement and clinical decision-makers to compare different novel therapies. The STC methodology enables population-matching across single-arm trials with imbalanced patient characteristics in small sample sizes.

Several studies have reported the relationship of age to response to treatment in aGVHD. One study of risk factors associated with aGVHD assessed day 28 ORR according to age, with a reference group of patients <20 year (n=421) compared with patients 21 to 40 years (n=459) and >40 years (n=843).^34^ The OR was 0.8 (95% CI, 0.6 to 1.1, *P*=0.1) for patients <20 years versus those 21 to 40 years, and 0.8 (95% CI, 0.6 to 1.1, *P*=0.15) for patients <20 years versus those >40 years. Likewise, another recent study reported outcomes of both adult and pediatric patients with steroid-refractory aGVHD (n=203).^35^ The median patient age was 35 years (range <1 to 75 years), with 30% <18 years, 33% 18 to 40 years, and 37% over 40 years of age. The day 28 ORR for treatment of steroid-refractory aGVHD was similar for the three age groups: 34% (95% CI, 23 to 48), 36% (95% CI, 25 to 49), and 43% (95% CI, 32 to 55), respectively. Regression analysis demonstrated that age (per increase/decade) had no impact on day 28 ORR (OR of 1.00; 95% CI, 0.76 to 1.32). Thus, among patients with aGVHD, and more specifically among those with steroid-refractory aGVHD, there does not appear to be a relationship between age and response to treatment.

There are several other clinical considerations relating to the comparison of remestemcel-L-rknd and ruxolitinib. While both remestemcel-L-rknd and ruxolitinib have reported a median duration of therapy of 46 days, there is potential for a much longer course of treatment with ruxolitinib, which has a reported range of four to 473 days, with taper starting on Day 180.^29,36^ In contrast, the maximum duration of therapy for remestemcel-L-rknd is unlikely to exceed 97 days for patients with a complete response who experience a flare before day 70. Moreover, as an IV therapeutic, remestemcel-L-rknd is likely to confer greater patient adherence and bioavailability relative to orally administered ruxolitinib, particularly in patients with gut GVHD who may have impaired gastrointestinal absorption.^3^

In this STC analysis of ruxolitinib versus remestemcel-L-rknd, treatment with remestemcel-L-rknd was associated with an improved day 28 ORR in the steroid-refractory aGVHD patient population, but the association was not statistically significant. In the subgroup of patients with more severe disease (grade III-IV), remestemcel-L-rknd was associated with a statistically significant higher likelihood of achieving a day 28 ORR relative to ruxolitinib.

In contrast to ruxolitinib, treatment with remestemcel-L-rknd was associated with fewer TEAEs across all 30 TEAEs identified for comparison (i.e., those for which there were frequencies reported in both trials being compared), with 17 out of 30 being significantly lower (*P*≤0.05). The analysis also demonstrated a lower discontinuation rate due to AE (15% versus 31%) with remestemcel-L-rknd compared to ruxolitinib. These lower rates of AEs with remestemcel-L-rknd may translate into lower costs and fewer days of hospitalization, which is a major contributor to the economic burden of steroid-refractory aGVHD.^14^

These estimates may be used to populate future cost-effectiveness analyses for pediatric patients with steroid-refractory aGVHD and support reimbursement decisions on the most efficient use of health-care resources across various treatment options.

### Limitations

The age range of patients in the remestemcel-L-rknd trial (pediatric) and in REACH-1 (youngest patient age 18 years) was different; thus, it was statistically unfeasible to adjust for age. Accordingly, we assumed that age would not alter the results. This assumption is based on literature demonstrating similar outcomes in pediatric and adult populations, approval of ruxolitinib by the FDA in a pediatric population (≥12 and <18 years old) despite the lack of pediatric patients in the pivotal trial, and the need for reimbursement decision-makers to compare therapies with the available evidence.

An important limitation is that small sample sizes, such as those in both the ruxolitinib and remestemcel-L-rknd trials, may reduce the statistical power of the post-hoc analysis to detect statistically significant differences between treatments. Even if the STC does not reduce effective sample size, the coefficient estimates are still based on small samples, potentially limiting the robustness of the estimates. Moreover, STC methods rely on mutually reported patient characteristics across studies to match patient populations, and results are susceptible to residual confounding if patient characteristics are unbalanced with respect to unmeasured treatment effect modifiers. As population-matching techniques were used in the STC, there is an assumption of conditional constancy of absolute effects.^31^ This indicates an assumption that all relevant prognostic variables and effect modifiers are known and that treatment effects are constant at any level of prognostic variables and effect modifiers.

Furthermore, covariate adjustment was unfeasible for safety outcomes, and only non-adjusted methods (ITC) were possible owing to the limited number of patients experiencing individual TEAEs. ITC analyses were conducted to demonstrate general crude comparisons, and results should be interpreted with caution as patient population differences could impact results. Given that patient baseline comorbidities were not available in the REACH-1 presentation of ruxolitinib data, it is uncertain whether some of the differences in AEs between the two trials related to underlying conditions that are more likely to be present in adult versus pediatric populations.

## CONCLUSION

In this STC, remestemcel-L-rknd was associated with significant improvements in day 28 ORR compared with ruxolitinib in patients with severe (grade III-IV) SR aGVHD and a non-significant improvement in the overall population. Remestemcel-L-rknd was associated with fewer all-grade TEAEs, including the majority reaching statistical significance, and lower discontinuation rates suggesting an improved safety and tolerability profile over ruxolitinib. These findings help facilitate future treatment comparisons and health economic analyses for reimbursement decision-makers in an area with limited evidence.
